# Significant contribution of the CmeABC Efflux pump in high-level resistance to ciprofloxacin and tetracycline in *Campylobacter jejuni* and *Campylobacter coli* clinical isolates

**DOI:** 10.1186/s12941-021-00439-6

**Published:** 2021-05-20

**Authors:** Saeed Sharifi, Bita Bakhshi, Shahin Najar-peerayeh

**Affiliations:** Department of Bacteriology, Faculty of Medical Sciences, Tarbiat Modares University, Jalal-Ale-Ahmad Ave, 14117-13116 Tehran, Iran

**Keywords:** *Campylobacter*, Antibiotic resistance, RAPD-PCR, Efflux pump, RT-PCR

## Abstract

**Background:**

*Campylobacter* resistance to antimicrobial agents is regarded as a major concern worldwide. The aim of this study was to investigate the expression of the CmeABC efflux pump and the RAPD-PCR pattern in drug-resistant *Campylobacter* isolates.

**Methods:**

A total of 283 stool specimens were collected from children under the age of five with diarrhea. The minimum inhibitory concentration (MIC) of tetracycline and ciprofloxacin was determined by broth microdilution method and E-test, respectively. Detection of tetracycline and ciprofloxacin determinants was done by amplification of *tetO* gene and PCR-sequencing of the *gyrA* gene. The *cmeABC* transcriptional expression was analyzed by Real-time (RT)-PCR. Clonal correlation of resistant strains was determined by RAPD-PCR genotyping.

**Results:**

Out of 283 fecal samples, 20 (7.02%) samples were positive for *Campylobacter* spp. Analysis of duplex PCR assay of the *cadF* gene showed that 737 and 461 bp amplicons were corresponding to *Campylobacter jejuni* and *Campylobacter coli,* respectively. All of the 17 phenotypically tetracycline-resistant *Campylobacter* isolates harbored the *tetO* gene. Also, four phenotypically ciprofloxacin-resistant *Campylobacter* isolates had a point mutation at codon 257 of the *gyrA* gene (ACA to ATA; Thr > Ile). High-level expression of the *cmeA* gene was observed in ciprofloxacin-resistant and high-level tetracycline-resistant *Campylobacter* isolates, suggesting a positive correlation between the *cmeA* gene expression level and tetracycline resistance level. Moreover, a statistically significant difference was observed in the *cmeA* gene expression between ciprofloxacin-resistant and ciprofloxacin-susceptible strains, which signifies the crucial contribution of the efflux pump in conferring multiple drug resistance phenotype among *Campylobacter* spp. RAPD analysis of *Campylobacter* isolates exhibited 16 different patterns. Simpsone`s diversity index of RAPD-PCR was calculated as 0.85, showing a high level of homogeneity among the population; however, no clear correlation was detected among tetracycline and/or ciprofloxacin resistant isolates.

**Conclusion:**

Significant contribution of the CmeABC efflux pump in conferring high-level resistance to tetracycline and ciprofloxacin was observed in *C. jejuni* and *C. coli* clinical isolates. The resistant phenotype is suggested to be mediated by CmeABC efflux pumps, the *tetO* gene, and point mutation of the *gyrA* gene. Genotyping revealed no clonal correlation among resistant strains, indicating distinct evolution of tetracycline and ciprofloxacin resistant genotypes among the isolates.

## Introduction

*Campylobacter* species are one of the most important enteric pathogens and a major cause of acute bacterial gastroenteritis in humans worldwide [1, 2]. Human campylobacteriosis is induced mainly by *Campylobacter jejuni* (about 90% of cases), or by *Campylobacter coli* [2]. *C. jejuni* and *C. coli* cause a wide range of diseases from self-limiting gastroenteritis to life-threatening bacteremia and enterocolitis. Furthermore, *Campylobacter* infection is associated with autoimmune diseases including Guillain-Barré syndrome (GBS) and Miller Fisher syndrome [3, 4]. There are high numbers of *C. jejuni* and *C. coli* as commensals in the intestines of a wide variety of animals, particularly sheep, cattle, and poultry, which are considered as potential reservoirs for contaminating vegetable, food, and water and thus the cause of human infections [5, 6].

*Campylobacter* spp. show a remarkable ability to resist a wide spectrum of antimicrobial agents, resulting in widespread nosocomial outbreaks throughout the world [7, 8]. Moreover, the widespread and empirical use of broad-spectrum antibiotics including erythromycin, ciprofloxacin, kanamycin, nalidixic acid, and chloramphenicol in human, as well as in agricultural and aquacultural settings, has led to the emergence of multidrug-resistant (MDR) *Campylobacter* spp., which poses a major challenge to clinical therapy and contributes significantly to the increased morbidity and mortality rates [9, 10].

TetO is a ribosomal protection protein which its mode of action is through the inhibition of tetracycline replacement on the ribosomal binding site [12–14]. The *tetO* gene is located on a transmissible plasmid, which may explain the distribution of tetracycline resistance determinant in *Campylobacter* spp. [11–13]. Macrolide resistance in *C. jejuni* and *C. coli* is associated with a point mutation in the *gyrA* gene at codon 275 [14–16], which is chromosomally located.

A *Campylobacter* multidrug efflux pump, named CmeABC, is attributed to the resistance nodulation division (RND) family of efflux transporters. This efflux pump is encoded by an operon of three genes located on the chromosome, and is related to the widely distributed antimicrobial resistance of various families in different *Campylobacter* isolates [14, 17, 18]. The CmeABC efflux pump contains three major protein: a periplasmic fusion protein (CmeA), an inner membrane drug transporter (CmeB), and an outer membrane protein (CmeC) [18]. It has been shown that the CmeABC efflux pump confers low-level macrolide resistance in *Campylobacter* spp. [19–21]. There are a few studies on the role of active efflux in conferring high-level tetracycline resistance in *Campylobacter* isolates [22, 23]. Moreover, elucidating the relationship between the CmeABC efflux pump and the emergence of tetracycline-resistant *C. jejuni* and *C. coli* strains is essential to better understand the role of this active efflux pump in the development of the multidrug resistance phenotype in *Campylobacter* strains.

The main aim of this study was to determine the expression level of *cmeA* from the CmeABC efflux pump in ciprofloxacin- and tetracycline-susceptible and -resistant *Campylobacter* isolates. Also, random amplified polymorphic DNA-polymerase chain reaction (RAPD-PCR) was used to determine the genetic relatedness of *Campylobacter* isolates.

## Materials and methods

### Ethics

The study was reviewed and approved by the Medical Ethics Committee of Tarbiat Modares University (Code: IR.MODARES.REC) before it began. All the participants signed and approved the informed consent form.

### Bacterial strains characterization

A total of 283 stool specimens were collected from hospitalized children under the age of five with diarrhea, fever, and abdominal cramping in three major children hospitals, based in Tehran, Iran. Diarrhea was characterized as more than three loose episodes/day [24] Each specimen was inoculated into 2 ml Cary-Blair transport medium and incubated at 25 °C for 48 h. The transport broth cultures were then plated on modified charcoal-cefoperazone-deoxycholate agar (mCCDA) and Brucella agar (Merck, Germany) and incubated at 42 °C for 18–24 h in a microaerophilic gas-pack jar system (Mitsubishi, Chemicals, Japan). Presumptive *Campylobacter* colonies were investigated by microscopy to identify slender, spiral, curved, and S-shaped rods with corkscrew-like motion. The hippurate hydrolysis test was used to distinguish *C. jejuni* from *C. coli*. Finally, a duplex PCR assay targeting *Campylobacter* fibronectin-binding adhesin (*cadF)* gene was used to confirm the identity of *C. coli* and *C. jejuni* isolates as described previously [25]. *C. jejuni* ATCC 29,428 and *C. coli* ATCC 43,478 were used as standard controls.

### Antimicrobial susceptibility assay

Antimicrobial susceptibility profiles of *Campylobacter* isolates were determined by Kirby-Bauer disk diffusion method using Mueller–Hinton agar supplemented with 5% defibrinated horse blood and 20 mg/L of β-NAD (MH-F) plates and incubated at 41 °C for 24 h at microaerobic conditions. The standard procedures of EUCAST were strictly followed throughout the testing process. *C. jejuni* ATCC 33,560 was used as quality control [26]. The antibiotics tested were as follows (μg/disc): tetracycline (30), minocycline (30), erythromycin (15), chloramphenicol (30), gentamicin (10), ciprofloxacin (5) and nalidixic acid (30). The interpretation of results of minocycline, chloramphenicol, gentamicin and nalidixic acid was performed according to CLSI interpretive criteria for *Enterobacteriaceae* [27].

### Minimum inhibitory concentration of tetracycline and ciprofloxacin

Minimum inhibitory concentration (MIC) of tetracycline was assessed by broth microdilution method using Mueller–Hinton broth supplemented with 5% lysed horse blood and 20 mg/L of β-NAD (MH-F broth) and incubated at 41 °C for 24 h at microaerobic conditions. Resistance breakpoints were specified in accordance with the guidelines of EUCAST. *Staphylococcus aureus* ATCC 29,213 (under standard conditions for staphylococci) was used as quality control. In addition, the MIC of ciprofloxacin was determined by E-test (Liofilchem, USA).

### Detection of tetracycline and ciprofloxacin resistance determinants

Amplification of *tetO* and *gyrA* genes was carried out with gene-specific primers, as previously described [11, 28], using Master Mix (Ampliqon, Denmark) on a Bio-Rad thermal cycler (BioRad, USA). Cycling conditions were as follows: an initial denaturation step (at 95 °C for 7 min), followed by 35 cycles of denaturation (at 95 °C for 30 s), annealing (at 58 °C for 30 s), extension (at 72 °C for 30 s), and a final extension step at 72 °C for 10 min.

For all phenotypically ciprofloxacin-resistant isolates, the quinolone resistance-determining region (QRDR) of the *gyrA* gene was amplified using specific primers, as previously described [28], to amplify the responsible point mutation at C257A (Thr-86-Lys). The PCR products were subjected to sequencing using ABI 3730X capillary sequencer (Genfanavaran, Macrogen, Seoul, Korea) after purification with QIAquick Gel Extraction Kit (Qiagen). The sequences were analyzed using CLC Sequence Viewer 8.0 and Gene runner Version 6.5.49.

### Real-time (RT)-PCR analysis of *cmeA* transcription

RT-PCR was conducted to determine the expression level of the conserved regions of *cmeA* in all phenotypically tetracycline-resistant isolates. Briefly, total RNA was extracted using a RNA Mini Kit (Favorgen, Taiwan) according to the manufacturer's instructions, and subjected to cDNA synthesis using a RevertAid™ First Stranded cDNA Synthesis Kit (Fermentas, Lithonia). The 16SrRNA gene was used as an endogenous control. The PCR reaction mixture contained 12.5 µL of SYBR Green Master Mix, 400 ng of template DNA, 0.25 µM of forward and reverse primers each, and 12 µL of nuclease-free water. RT-PCR cycling conditions were as follows: 10 min at 50 °C, 5 min at 60 °C, and 5 min at 95 °C and then 40 cycles of 10 s at 95 °C, 30 s at 58 °C, and 15 s at 72 °C [29, 30].

### Random amplified polymorphic DNA (RAPD)-PCR of tetracycline-resistant isolates

RAPD-PCR was performed for tetracycline-resistant isolates as described previously [31, 32]. A 10-mer oligonucleotide primer (5′- GTGGATGCGA-3′) was used to characterize the isolates. PCR amplification was performed based on the following program: one cycle of 4.5 min at 94 °C, followed by 5 low-stringency cycles of 30 s at 94 °C, 2 min at 26 °C, and 2 min at 72 °C, and then 35 high-stringency cycles of 30 s at 94 °C, 1 min at 45 °C, and 2 min at 72 °C. The cycling was completed with a cycle of 5 min at 72 °C, and the reaction products were stored at 4 °C prior to analysis. Amplification products were detected by agarose gel electrophoresis with ethidium bromide staining and recorded by a gel documentation system (MWG-Biotech, Germany). Cluster analysis was done using GelCompar II software (Applied Maths, Belgium).

### Statistical analysis

The correlation between the *cmeA* gene expression level and tetracycline-resistance level was determined by Pearson correlation coefficient test. Comparisons between ciprofloxacin-resistant and ciprofloxacin susceptible *Campylobacter* isolates regarding the *cmeA* gene expression level was performed with T-test. All experiments were done in triplicates, and the data were expressed as the mean ± standard deviation (SD). Mean differences were considered as statistically nonsignificant when the *p* values were > 0.05.

## Results

### Bacterial strains

**Fig. 1:**
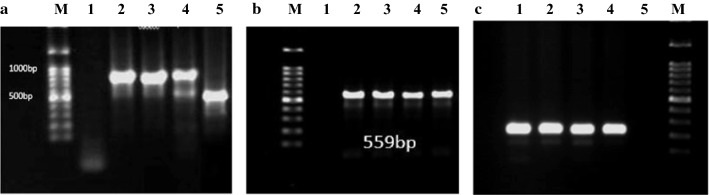
**a** Duplex PCR assay of *cadF* gene. Lane M, 100 bp DNA ladder; lane 1, negative control; lane 2, positive control (*C. jejuni* ATCC 29,428); lane 3–4, clinical *C. jejuni* strains (737 bp); lane 5, positive control (*C. coli* ATCC 43,478) (461 bp). **b** Analysis of the PCR assay of *tetO* (559 bp) gene product. Lane M, 100 bp DNA ladder; lane 1, negative control; lane 2, positive control; lane 3–5, *tetO* gene in tetracycline resistant isolates. **c** Analysis of PCR assay of the *gryA* (220 bp) gene product. Lane M: 100 bp DNA ladder; Lane 1–3: the *gyrA* gene in ciprofloxacin resistant isolates; Lane 4: positive control; Lane 5: negative control

In this study, 20 *Campylobacter* isolates were collected from stool samples of children with diarrhea. Among the 20 isolates, 18 and 2 isolates were identified as *C. jejuni* and *C. coli*, respectively*,* based on the hippurate hydrolysis test and duplex PCR assay of the *cadF* gene (Fig. 1a), and as expected, both methods (biochemical and molecular) produced the same results.

### Antimicrobial susceptibility testing

According to MIC results, 17 (2 *C. coli* and 15 *C. jejuni*) out of 20 isolates (85%) were resistant to tetracycline (MIC: 4–64 µg/mL). The tetracycline MIC of 64 µg/mL was observed in 2 isolates (10%), 32 µg/mL in 2 isolates (10%), 16 µg/mL in 4 isolates (20%), 8 µg/mL in 1 isolate (5%), and 4 µg/mL in 8 isolates (40%). Also, 3 out of 20 isolates (15%) were sensitive to tetracycline (MIC ≤ 0.5 µg/mL).

The isolates showed lower resistance to ciprofloxacin, minocycline, and nalidixic acid (20% for each). The MIC results of ciprofloxacin-resistant isolates (n = 4) showed a high level of resistance (MIC ≥ 32 µg/mL) to ciprofloxacin as determined by E-test. In this study, two *C. coli* strains were identified which were resistant to tetracycline and susceptible to ciprofloxacin. No isolate was resistant to chloramphenicol, gentamicin, and erythromycin.

### Molecular characterization of antimicrobial resistance

**Fig. 2:**
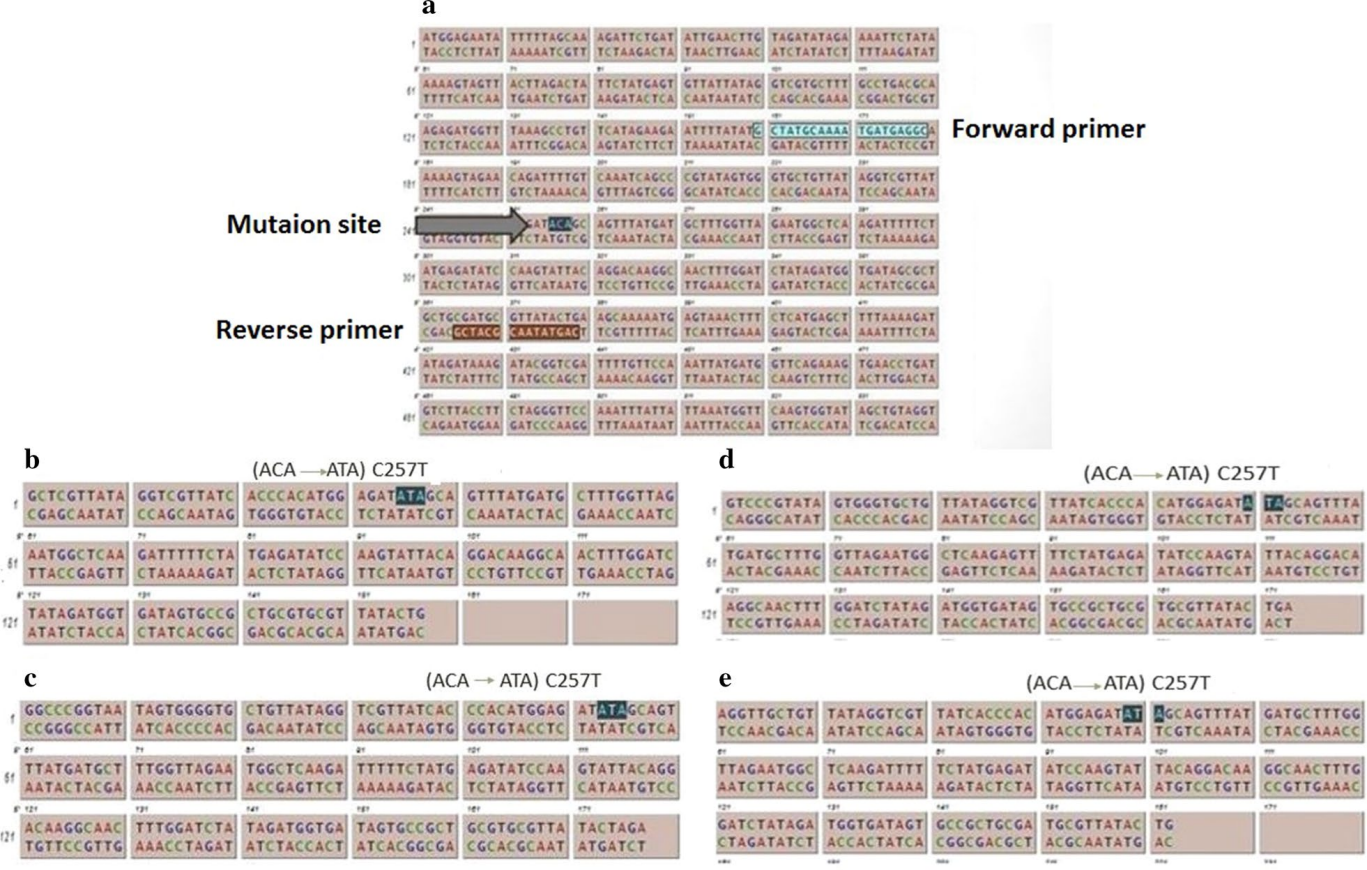
Analysis of sequencing result of the *gryA* gene product. **a** Sequencing result of the *gryA* gene in *C. jejuni* ATCC 33,560; **b**–**e**: four ciprofloxacin resistant isolates

Confirming the antibiotic sensitivity testing, out of the 20 isolates, the *tetO* gene was detected in 17 (85%) cases. The *tetO* gene was not found in the three tetracycline-sensitive isolates, as determined by MIC test (Fig. 1b). The *gyrA* gene of all ciprofloxacin-resistant isolates was amplified and sequenced (Fig. 1c). The obtained results showed the common point mutation “ACA” to “ATA” at C257T (Thr-86-Ile) in all ciprofloxacin-resistant isolates (Fig. 2).

### Quantification of *cmeA* expression

**Fig. 3:**
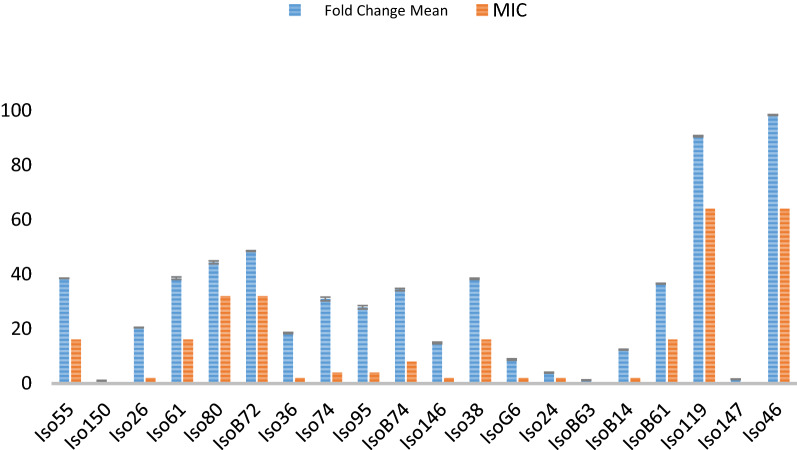
RT-PCR analysis of the expression of the *cmeA* gene in *Campylobacter* isolates. Relative expression level of *cmeA* in *Campylobacter* isolates in relation to MIC. The values were presented as the mean ± SD of at least two independent experiments

The expression of the *cmeA* gene was quantitatively assessed using RT-PCR. As shown in Fig. 3, the expression of *cmeA* in two high tetracycline-resistant *Campylobacter* isolates (MIC of 64 µg/mL) was 90–98 fold higher than in tetracycline-susceptible isolates (*p*-value ≤ 0.05). Similarly, the expression of *cmeA* in two tetracycline-resistant isolates with MIC of 32 µg/mL was 44–48 fold higher than in tetracycline-susceptible isolates (*p*-value ≤ 0.05). Moreover, the expression of *cmeA* in four isolates with tetracycline MIC of 16 µg/mL and one isolate with MIC of 8 µg/mL was 36–38 and 34 fold higher than in tetracycline-susceptible isolates, respectively (*p*-value ≤ 0.05), and eight isolates with MIC of 4 µg/mL showed 3–30 folds increase in *cmeA* expression compared with susceptible strains. Statistically, a positive correlation was detected between tetracycline resistance level and the *cmeA* gene expression level (correlation coefficient = 0.952).

**Fig. 4:**
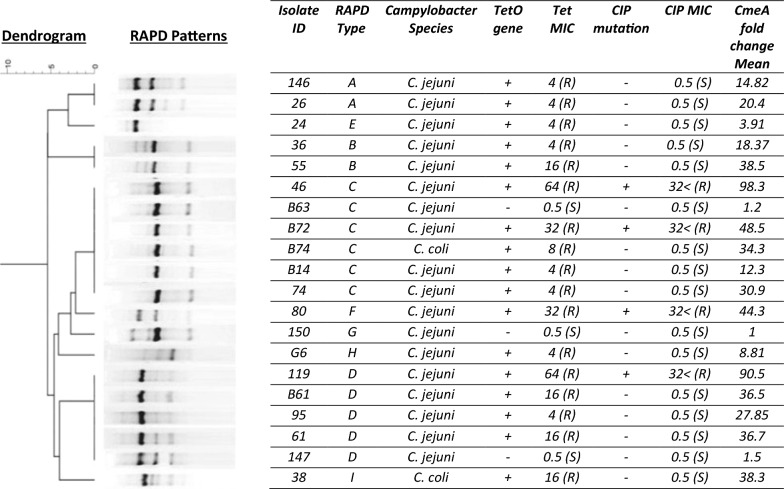
Dendrogram based on the hierarchic numerical analysis of the RAPD-PCR profiles obtained for 20 *Campylobacter* isolates, employing the Pearson correlation coefficient and UPGMA for clustering. Dendrogram is shown in relation to resistance properties, sequence data, and *cmeA* expression properties

The level of *cmeA* gene expression in ciprofloxacin susceptible strains (n = 16) (MIC = 0.5) ranged from 1 to 38.5 folds, while its expression in ciprofloxacin resistant strains (n = 4) (MIC =  < 32) ranged from 44 to 98 folds (Fig. 4). A statistically significant difference was observed in the *cmeA* gene expression level between ciprofloxacin resistant and ciprofloxacin susceptible strains (*p*-value ≤ 0.05).

### RAPD- PCR genotyping

The genetic diversity among *Campylobacter* isolates was investigated by RAPD-PCR. The dendrogram derived from the RAPD-PCR profiles is shown in Fig. 4. Based on the genotyping, the tetracycline-resistant *Campylobacter* isolates were divided into the following types: type A, two isolates; type B, two isolates; type C, six isolates; type D, five isolates; and types E, F, G, H, and I, one isolate each.

## Discussion

*Campylobacter* is considered as the most important causative agent of bacterial gastroenteritis in young children, but only a limited number of studies have focused on antibiotic resistance in *Campylobacter* strains from children, especially in Iran. Therefore, there is a great demand for research and evidence on the burden of antibiotic-resistant *Campylobacter* strains on pediatric population. Typically, *C. jejuni* enteritis is usually mild and self-limiting; however, in more complicated cases, including systemic infections, neuropathy diseases (e.g. Guillain–Barre Syndrome), and severe or long-lasting infections, it is necessary to administer antimicrobial treatment [23]. The most common antimicrobial agents used for the treatment of campylobacteriosis are macrolides, such as erythromycin, and fluoroquinolones, such as ciprofloxacin [23], which are suitable for empirical treatment; however, tetracycline is used as an alternative drug [33, 34]. All *Campylobacter* spp. were found to be susceptible to erythromycin, gentamicin, and chloramphenicol; however, maximum antimicrobial resistance was observed to tetracycline (85%) and the second most resistance was seen to ciprofloxacin (20%). Therefore, the present study findings, together with previous reports, support the continued use of erythromycin as first-line therapy for campylobacteriosis in Iran.

In this study, the antibiotic susceptibility of *Campylobacter* strains isolated from children was investigated based on EUCAST recommendations. The highest resistance rate was observed to tetracycline. Resistance to tetracycline was shown here to be increased compared to previous studies on chicken carcasses, food samples and patients with diarrhea in Iran [35, 36]. Albert [37] and Gallay et al*.* [38] revealed that resistance to tetracycline has increased among diarrheal patients and domestic animals, since 1999. These results may not be unexpected, since *C. jejuni* is a zoonotic pathogen with a broad animal reservoir, and the use of medication in both animal agriculture and human could influence the progression of antimicrobial resistance. It has been demonstrated that the high rate of tetracycline resistance is associated with the transmissible plasmid-encoded *tetO* gene [39]. Confirming this, the *tetO* gene was observed in all tetracycline-resistant *Campylobacter* isolates in the present study. It is worth noting that the level of resistance to tetracycline was variable among resistant *Campylobacter* isolates, emphasizing the potential contribution of other mechanisms. Antimicrobial resistance in *Campylobacter* strains is often interceded by multiple mechanisms [34]; and one of the major mechanisms of resistance to antimicrobials in *C. jejuni* strains is the CmeABC efflux pump [33]. CmeABC plays a key role in mediating resistance to antibiotics, bile salts, and some disinfectants [40]. The expression of the *cmeA* gene was statistically correlated with high level resistance to tetracycline in the present study, indicating that the efflux pump plays an important accessory role in mediating resistance to tetracycline among *Campylobacter* isolates [9]. Gibreel et al. discovered that tetO-mediated tetracycline resistance in *C. jejuni* isolates is completely abolished if the CmeABC efflux pump is inactive, which seems to be due to the cells being flooded with tetracycline and completely overwhelming the ribosomal protection provided by TetO protein. This highlights the major contribution of the CmeABC efflux pump to the acquired tetracycline resistance in *C. jejuni* [23].

In the present study, resistance to ciprofloxacin was found in 20% of samples, which shows an increase in comparison to a previous study [41], while depicts a decrease compared to another report from Iran in 2011 [42]. Lehtopolku et al. (2010) revealed that resistance to ciprofloxacin among 1808 isolates was high (80%) [43]. However, Oza et al. (2003) showed the lowest resistance to ciprofloxacin (3%), indicating controversial reports in different populations [44]. Excessive use of fluoroquinolones such as ciprofloxacin for the treatment of campylobacteriosis may be the reason for the emergence of high ciprofloxacin-resistant *Campylobacter* isolates [45]. It has been documented that a point mutation in *gyrA* is related to ciprofloxacin resistance [46]. A point mutation at codon 257(ACA → ATA) of the *gyrA* gene was found in all ciprofloxacin-resistant *Campylobacter* isolates, which results in a Thr-86-Ile change in the GyrA protein. This mutation is the most commonly observed mutation in fluoroquinolone-resistant *Campylobacter* isolates and results in a ciprofloxacin MIC ≥ 16 μg/mL. Indeed, all ciprofloxacin-resistant *Campylobacter* isolates in this study showed MIC ≥ 32 μg/mL, which is consistent with the Thr-86-Ile change in GyrA. The Thr-86-Lys and Asp-90-Asn mutations, which are less common, were not detected in this study. These latter mutations are associated with intermediate-level fluoroquinolone resistance [47].

The level of *cmeA* gene expression in strains with both ciprofloxacin and tetracycline resistance was 44–98 folds higher than strains with ciprofloxacin and tetracycline susceptibility. This signifies the crucial contribution of *cmeA* expression in pumping ciprofloxacin and tetracycline out of the bacterial cell. A previous study by Cagliero et al. (2007) showed that in *cmeB*-inactivated mutants, MICs of all antibiotics, including ciprofloxacin, were considerably reduced, indicating the direct involvement of the CmeABC efflux system in inducing multidrug resistance [33]. Interestingly, in our collection, the high level of *cmeA* expression was observed in four strains with high levels of resistance to tetracycline and ciprofloxacin, which implies that *cmeA* is one the most important determinants of multiple resistance in *Campylobacter* strains.

RAPD-PCR results showed that tetracycline-resistant *Campylobacter* isolates distributed in different RAPD types which suggests that they are unrelated genotypes. Moreover, no genotyping linkage was detected among ciprofloxacin or tetracycline resistant strains, which implies the independent evolution of resistance genotypes.

In conclusion, these results provide evidence of high-level resistance of *Campylobacter* isolates to tetracycline and ciprofloxacin, which is mediated by CmeABC efflux pumps in parallel with plasmid-encoded *tetO* gene as well as a point mutation in the *gyrA* gene, respectively. We maintain pessimism that no clear clonal correlation or genotyping profile among multidrug-resistant *Campylobacter* isolates in human samples reveals their potential self-governing and distinct evolutionary emergence.

## Data Availability

The datasets of the current study are available within the article or could be obtained from the corresponding author upon request.
